# Impact of Cancer, Inflammation, and No Standard Risk Factors in Patients With Myocardial Infarction

**DOI:** 10.1016/j.jacasi.2024.03.008

**Published:** 2024-05-28

**Authors:** Hiroaki Yaginuma, Yuichi Saito, Hiroki Goto, Kazunari Asada, Yuki Shiko, Takanori Sato, Osamu Hashimoto, Hideki Kitahara, Yoshio Kobayashi

**Affiliations:** a Department of Cardiovascular Medicine, Chiba University Hospital, Chiba, Japan; b Biostatistics Section, Clinical Research Center, Chiba University Hospital, Chiba, Japan; c Department of Cardiology, Chiba Emergency and Psychiatric Medical Center, Chiba, Japan

**Keywords:** acute myocardial infarction, cancer, cardiovascular risk factors, inflammatory disease, prognosis

## Abstract

**Background:**

The lack of standard modifiable cardiovascular risk factors (SMuRFs), including hypertension, diabetes, dyslipidemia, and smoking, is reportedly associated with poor outcomes in acute myocardial infarction (AMI). Among patients with no SMuRFs, cancer and chronic systemic inflammatory diseases (CSIDs) may be major etiologies of AMI.

**Objectives:**

The purpose of this study was to evaluate clinical characteristics and outcomes of patients with cancer, CSIDs, and no SMuRFs in AMI.

**Methods:**

This multicenter registry included 2,480 patients with AMI undergoing percutaneous coronary intervention. Patients were divided into 4 groups: active cancer, CSIDs, no SMuRFs, and those remaining. The coprimary endpoint was major adverse cardiovascular events (MACE) and major bleeding events, during hospitalization and after discharge.

**Results:**

Of 2,480 patients, 104 (4.2%), 94 (3.8%), and 120 (4.8%) were grouped as cancer, CSIDs, and no SMuRFs, respectively. During the hospitalization, MACE rates were highest in the no SMuRFs group, followed by the cancer, CSIDs, and SMuRFs groups (22.5% vs 15.4% vs 12.8% vs 10.2%; *P* < 0.001), whereas bleeding risks were highest in the cancer group, followed by the no SMuRFs, CSIDs, and SMuRFs groups (15.4% vs 10.8% vs 7.5% vs 4.9%; *P* < 0.001). After discharge, the rates of MACE (33.3% vs 22.7% vs 11.3% vs 9.2%; *P* < 0.001) and bleeding events (8.6% vs 6.7% vs 3.8% vs 2.9%; *P =* 0.01) were higher in the cancer group than in the CSIDs, no SMuRFs, and SMuRFs groups.

**Conclusions:**

Patients with active cancer, CSIDs, and no SMuRFs differently had worse outcomes after AMI in ischemic and bleeding endpoints during hospitalization and/or after discharge, compared with those with SMuRFs.

Atherosclerotic cardiovascular diseases, such as acute myocardial infarction (MI), are mainly attributable to standard modifiable cardiovascular risk factors (SMuRFs), including hypertension, diabetes, dyslipidemia, and smoking, across the world.^1^ In patients with MI, the identification and targeted strategies against SMuRFs contribute to a reduction in the burden of cardiovascular events.^2^ However, it has been reported that a significant proportion of patients with acute MI have no SMuRFs,^3^^,^^4^ and the lack of SMuRFs is counterintuitively associated with worse clinical outcomes in a setting of acute MI.^5^, ^6^, ^7^, ^8^ Patients with acute MI and no SMuRFs should have nonstandard cardiovascular risk factors such as disorders in sleep, nutrition, physical activity, mental and oral health, coagulation system, and genetics, among which active cancer and inflammatory diseases may be major etiologies of MI.^3^ Our previous study showed that approximately one-third of patients with acute MI and no SMuRFs had active cancer and chronic systemic inflammatory diseases (CSIDs), including rheumatoid arthritis and systemic lupus erythematosus, as potential underlying risk factors for MI.^9^ Given that the presence of active cancer and CSIDs is independently associated with a worse prognosis in patients with MI in previous reports,^10^, ^11^, ^12^, ^13^, ^14^ whether SMuRF-less patients without active cancer and CSIDs have an increased risk of cardiovascular and bleeding events after acute MI remains uncertain. In the present study, we evaluated the clinical characteristics and outcomes of acute MI patients with active cancer, CSIDs, and no SMuRFs.

## Methods

### Study design

This was a retrospective, multicenter registry study. Between January 2012 and December 2021, a total of 2,485 patients with acute MI, including both ST-segment elevation and non–ST-segment elevation MI, underwent primary percutaneous coronary intervention (PCI) at 4 centers (Chiba University Hospital, Eastern Chiba Medical Center, Chiba Emergency Medical Center, and Chiba Aoba Municipal Hospital) in Japan. Acute MI was defined by the Fourth Universal Definition of MI.^15^ All PCI procedures were performed per local standard practice with the predominant use of dual antiplatelet therapy, intracoronary imaging, and contemporary drug-eluting stents.^16^, ^17^, ^18^, ^19^, ^20^, ^21^, ^22^ This study was conducted in accordance with the Declaration of Helsinki and was approved by the ethics committees of each center. Informed consent for this study was ascertained in the form of an opt-out.

### Definitions of active cancer, CSID, and SMuRF

In the present study, patients who were planned to undergo cancer surgery; were receiving anticancer drugs and radiotherapy; and had recurrent, metastatic, and/or inoperable cancer were defined as having active cancer.^10^ CSIDs were defined with the diagnosis of systemic inflammatory disorders including connective tissue (rheumatoid arthritis, systemic lupus erythematosus, and so on) and organ-specific (inflammatory bowel diseases such as ulcerative colitis and Crohn's disease, psoriasis, and so on) diseases as previously reported.^23^, ^24^, ^25^, ^26^, ^27^ Patients with CSIDs may have undergone specific medical therapies. Patients with both CSIDs and active cancer (n = 5) were excluded. Thus, 2,480 patients with acute MI undergoing PCI were eventually included in the current analysis.

SMuRFs included hypertension, diabetes, dyslipidemia, and current smoking in this study.^9^ Hypertension was defined as having a previous diagnosis of hypertension or previous antihypertensive pharmacological treatment, or a new diagnosis of hypertension during hospitalization with systolic blood pressure ≥140 mm Hg and/or diastolic blood pressure ≥90 mm Hg. Diabetes was defined as previous diagnosis, previous glucose-lowering medications, or hemoglobin A1c ≥6.5%. Dyslipidemia was defined as previous diagnosis, previous pharmacological treatment, low-density lipoprotein cholesterol ≥140 mg/dL, high-density lipoprotein cholesterol <40 mg/dL, or fasting triglycerides >150 mg/dL. Current smoking was defined as a history of tobacco smoking within the past year.^28^ SMuRF-less patients were defined as those having none of the 4 cardiovascular risk factors, whereas patients with SMuRFs were determined as having at least 1 of the risk factors. In the present study, patients were divided into 4 groups: active cancer; CSIDs; no SMuRFs; and those remaining (SMuRFs group).

### Outcomes

Follow-up data were obtained from medical records at the 4 centers. The coprimary endpoint of the present study included major adverse cardiovascular events (MACE) and major bleeding events (Bleeding Academic Research Consortium type 3 or 5) during hospitalization for acute MI and after discharge.^29^ MACE was defined as a composite of all-cause death, recurrent MI, and ischemic stroke according to the consensus documents.^30^

### Statistical analysis

Statistical analysis was conducted using JMP Pro 16 software (SAS Institute). Data are expressed as mean ± SD, median (IQR), or frequencies (percentages) as appropriate. Continuous variables were assessed with analysis of variance or the Kruskal-Wallis test, and categorical variables were compared using the Fisher exact test. The Kaplan-Meier analysis was performed to calculate the time to MACE and major bleeding events after discharge with landmark analysis using the date of discharge as a landmark, excluding patients who died during the index hospitalization for acute MI and had no follow-up information. Multivariable analysis was performed using logistic regression and the Cox proportional hazard model to estimate unadjusted and adjusted HRs with corresponding 95% CIs of MACE and major bleeding events during the index hospitalization and after discharge. In addition to age and sex, the study groups (the main interest of this study) and the presence of cardiogenic shock, which is well-known to be associated with in-hospital mortality in a setting of acute MI,^31^ were included in the multivariable analysis. Therapeutic approaches that would be associated with ischemic and bleeding events, including the use of drug-eluting stents, oral anticoagulation, and statins, were also included in the multivariable models. Because medications at discharge were available in the present study, they were not included in the multivariable logistic regression analysis for in-hospital outcomes. Sensitivity analysis was performed for bleeding outcomes after discharge using Fine and Gray subdistribution hazard models considering death as a competing risk. A *P* value <0.05 was considered statistically significant.

## Results

Of the 2,480 patients, 104 (4.2%), 94 (3.8%), and 120 (4.8%) were grouped as active cancer, CSIDs, and no SMuRFs. Details of active cancer and CSIDs are listed in Supplemental Tables S1 and S2. Colon cancer was the most common malignancy, followed by blood, lung, and liver cancers (Supplemental Table S1), while rheumatoid arthritis was the leading etiology of CSIDs (Supplemental Table S2). Overall, 142 of 2,480 (5.7%) patients had no SMuRFs, among whom 22 (15.5%) had either active cancer or CSIDs. In the active cancer and CSIDs groups, 14 of 104 (13.5%) and 8 of 94 (8.5%) patients had no SMuRFs, respectively. Baseline patient characteristics are shown in Table 1. The mean age was highest in the active cancer group, followed by the no SMuRFs, SMuRFs, and CSIDs groups, and the proportion of women was highest in the CSIDs group (Table 1). Cardiogenic shock and cardiac arrest were most frequently observed in the no SMuRFs group (Table 1).

**Table 1:** Baseline Patient Characteristics

	All (N = 2,480)	SMuRFs (n = 2,162)	No SMuRFs (n = 120)	Active Cancer (n = 104)	CSIDs (n = 94)	*P* Value
Age, y	68.0 ± 12.6	67.7 ± 12.6	69.1 ± 14.9	71.7 ± 8.9	67.3 ± 12.7	0.01
Men	1,909 (77.0)	1,678 (77.6)	94 (78.3)	82 (78.9)	55 (58.5)	<0.001
Body mass index, kg/m^2^	24.3 ± 3.9	24.5 ± 3.5	23.0 ± 3.5	23.1 ± 3.7	23.3 ± 3.5	<0.001
Hypertension	1,695 (68.4)	1,560 (72.2)	0 (0)	71 (68.3)	64 (68.1)	<0.001
Diabetes	920 (37.1)	854 (39.5)	0 (0)	39 (37.5)	27 (28.7)	<0.001
Dyslipidemia	1,563 (63.0)	1,456 (67.4)	0 (0)	50 (48.0)	57 (60.6)	<0.001
Current smoker	886 (35.7)	838 (38.8)	0 (0)	24 (23.0)	24 (25.5)	<0.001
Prior myocardial infarction	196 (7.9)	177 (8.2)	3 (2.5)	11 (10.6)	5 (5.3)	0.054
Atrial fibrillation	146 (5.9)	127 (5.9)	11 (9.2)	5 (4.8)	3 (3.2)	0.31
History of heart failure	59 (2.4)	53 (2.5)	2 (1.7)	1 (1.0)	3 (3.2)	0.76
eGFR, mL/min/1.73 m^2^	64.0 ± 24.4	64.3 ± 24.5	61.5 ± 17.7	60.1 ± 26.7	65.1 ± 26.9	0.22
LVEF, %	47.2 ± 13.1	47.1 ± 12.9	45.9 ± 15.3	46.3 ± 14.8	50.5 ± 12.9	0.07
Clinical presentation						
STEMI	1,737 (70.0)	1,524 (70.5)	90 (75.0)	64 (61.5)	59 (62.8)	0.06
NSTEMI	743 (30.0)	638 (29.5)	30 (25.0)	40 (38.5)	35 (37.2)	0.06
Cardiogenic shock	404 (16.3)	341 (15.8)	32 (26.7)	18 (17.3)	13 (13.8)	0.02
Cardiac arrest	264 (10.7)	220 (10.2)	30 (25.0)	7 (6.7)	7 (7.5)	<0.001
Mechanical circulatory support						
IABP	279 (11.3)	238 (11.0)	22 (18.3)	10 (9.6)	9 (9.6)	0.11
ECMO	123 (5.0)	97 (4.5)	17 (14.2)	4 (3.9)	5 (5.3)	<0.001
Intracoronary imaging	2,428 (97.9)	2,124 (98.2)	116 (96.7)	99 (95.2)	89 (94.7)	0.01
Drug-eluting stent	2,255 (90.9)	1,990 (92.0)	107 (89.2)	77 (74.0)	81 (86.2)	<0.001

Values are mean ± SD or n (%).

CSID = chronic systemic inflammatory disease; ECMO = extracorporeal membrane oxygenation; eGFR = estimated glomerular filtration rate; IABP = intra-aortic balloon pumping; LVEF = left ventricular ejection fraction; NSTEMI = non–ST-segment elevation myocardial infarction; SMuRF = standard modifiable cardiovascular risk factor; STEMI = ST-segment elevation myocardial infarction.

During hospitalization for acute MI, 276 (11.1%) patients had MACE with all-cause death in 223 (9.0%), recurrent MI in 31 (1.3%), and ischemic stroke in 50 (2.0%), while 141 (5.7%) patients experienced major bleeding events (Table 2). The incidence of in-hospital MACE was highest in the no SMuRFs group, followed by the active cancer, CSIDs, and SMuRFs groups (Table 2). The rate of in-hospital major bleeding events was highest in the active cancer group, followed by the no SMuRFs, CSIDs, and SMuRFs groups (Table 2). Multivariable analysis identified older age, cardiogenic shock, and no SMuRFs as factors significantly associated with in-hospital MACE (Table 3), and cardiogenic shock, active cancer, and no SMuRFs related to major bleeding events during hospitalization (Table 4).

**Table 2:** In-Hospital Clinical Outcomes

	All (N = 2,480)	SMuRFs (n = 2,162)	No SMuRFs (n = 120)	Active Cancer (n = 104)	CSIDs (n = 94)	*P* Value
MACE	276 (11.1)	221 (10.2)	27 (22.5)	16 (15.4)	12 (12.8)	<0.001
All-cause death	223 (9.0)	175 (8.1)	25 (20.8)	13 (12.5)	10 (10.6)	<0.001
Myocardial infarction	31 (1.3)	28 (1.3)	0 (0)	2 (1.9)	1 (1.1)	0.56
Ischemic stroke	50 (2.0)	42 (1.9)	2 (1.7)	4 (3.9)	2 (2.1)	0.52
Major bleeding events	141 (5.7)	105 (4.9)	13 (10.8)	16 (15.4)	7 (7.5)	<0.001

Values are n (%).

MACE = major adverse cardiovascular event(s); other abbreviations as in Table 1.

**Table 3:** Logistic Regression Analysis for In-Hospital MACE

	Univariable		Multivariable	
	OR (95% CI)	*P* Value	OR (95% CI)	*P* Value
Age, y	1.02 (1.01-1.03)	0.004	1.02 (1.01-1.03)	<0.001
Men	0.95 (0.71-1.27)	0.71	1.01 (0.72-1.42)	0.95
Cardiogenic shock	12.97 (9.84-17.11)	<0.001	13.30 (10.03-17.63)	<0.001
Drug-eluting stent	0.80 (0.53-1.20)	0.27	0.90 (0.57-1.44)	0.67
No SMuRFs group	2.50 (1.68-3.78)	<0.001	2.14 (1.33-3.45)	0.002
Active cancer group	1.48 (0.86-2.56)	0.16	1.38 (0.74-2.58)	0.31
CSIDs group	1.18 (0.63-2.19)	0.61	1.34 (0.66-2.73)	0.42

Abbreviations as in Tables 1 and 2.

**Table 4:** Logistic Regression Analysis for In-Hospital Major Bleeding Events

	Univariable		Multivariable	
	OR (95% CI)	*P* Value	OR (95% CI)	*P* Value
Age, y	1.01 (0.99-1.02)	0.34	1.01 (0.99-1.02)	0.47
Men	0.80 (0.55-1.18)	0.25	0.78 (0.51-1.20)	0.26
Cardiogenic shock	9.94 (6.94-14.22)	<0.001	10.16 (7.05-14.63)	<0.001
Drug-eluting stent	0.70 (0.41-1.19)	0.19	0.92 (0.52-1.64)	0.78
No SMuRFs group	2.61 (1.54-4.43)	<0.001	2.02 (1.14-3.57)	0.02
Active cancer group	3.27 (1.87-5.75)	<0.001	3.53 (1.88-6.65)	<0.001
CSIDs group	1.35 (0.62-2.98)	0.45	1.51 (0.64-3.58)	0.35

Abbreviations as in Table 1.

**Figure 1:**
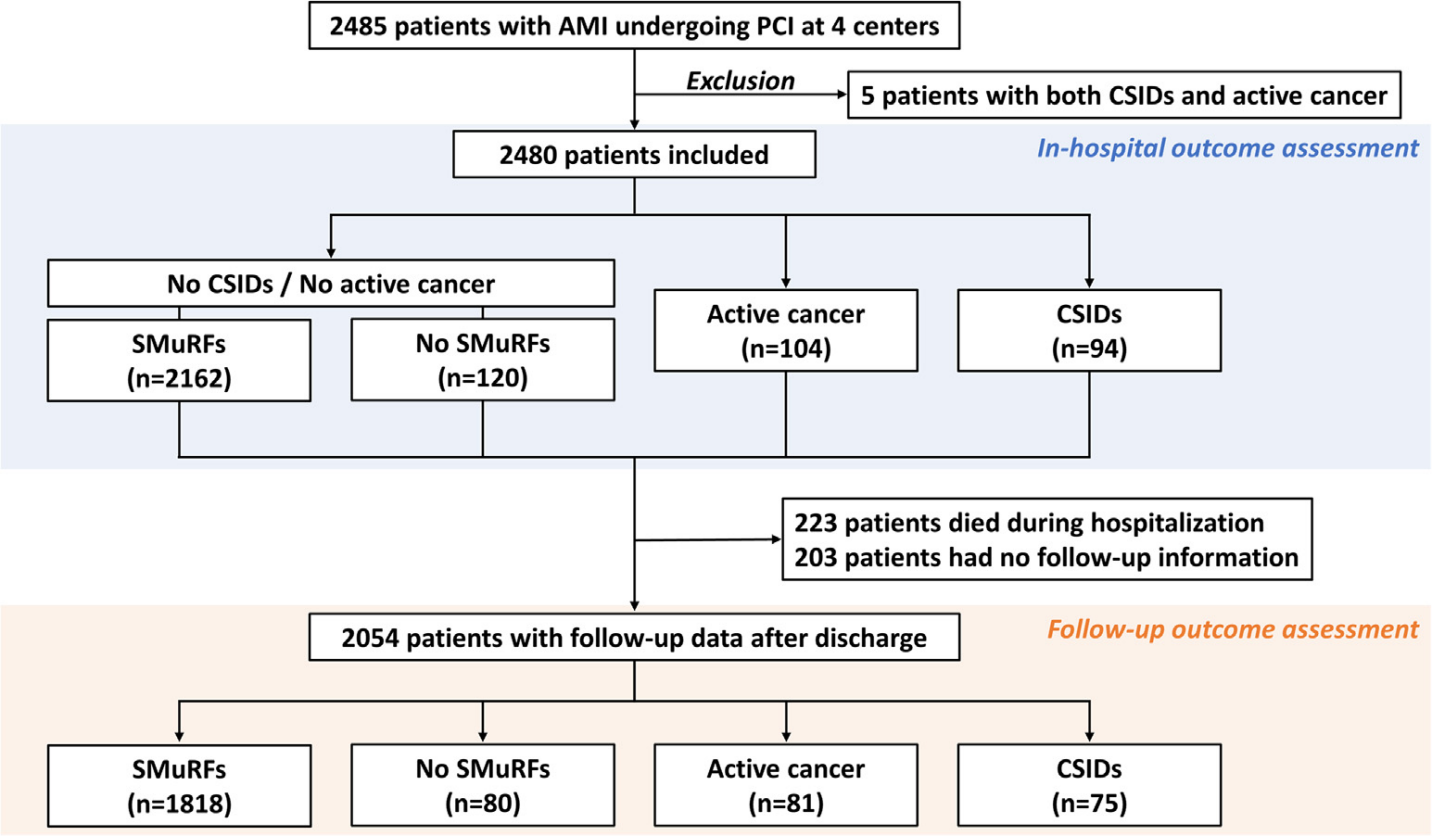
Study Flow In-hospital clinical outcomes were assessed in the active cancer, chronic systemic inflammatory diseases (CSIDs), no standard modifiable cardiovascular risk factors (SMuRFs), and SMuRFs groups. Outcomes after discharge were also evaluated in patients who survived to discharge and had follow-up information. AMI = acute myocardial infarction; PCI = percutaneous coronary intervention.

Among 2,257 patients who survived to discharge, 203 had no follow-up information after discharge. Thus, follow-up outcomes were assessed in 2,054 patients (Figure 1). Medications at discharge are listed in Table 5. During the median follow-up of 539 days (Q1, Q3: 349, 1,313 days) after discharge, 220 of 2,054 (10.7%) patients developed MACE. The incidence of MACE after discharge was highest in the active cancer group, followed by the CSIDs, no SMuRFs, and SMuRFs groups (Table 6). The Kaplan-Meier analysis demonstrated that patients with active cancer had an increased risk of MACE after discharge than other groups, mainly driven by a higher risk of all-cause death (Central Illustration, Table 6). The risk of recurrent MI did not differ significantly among the 4 groups, while the incidence of ischemic stroke was highest in the CSIDs group, followed by the active cancer, no SMuRFs, and SMuRFs groups (Table 6). The risk of major bleeding events after discharge was higher in the active cancer group than in other groups (Central Illustration, Table 6). The Cox proportional hazards analysis showed that older age, drug-eluting stents, active cancer, and CSIDs were significantly associated with an increased risk of MACE (Table 7), and that older age, female gender, and active cancer were related to a higher bleeding risk after discharge (Table 8). Competing risk analysis showed similar results in bleeding events after discharge (Supplemental Table S3).

**Table 5:** Medications at Discharge

	All (N = 2,054)	SMuRFs (n = 1,818)	No SMuRFs (n = 80)	Active Cancer (n = 81)	CSIDs (n = 75)	*P* Value
Antiplatelet therapy	2,026 (98.6)	1,798 (98.9)	77 (96.3)	77 (95.1)	74 (98.7)	0.01
Aspirin	1,898 (92.4)	1,682 (92.5)	74 (92.5)	69 (85.2)	73 (97.3)	0.04
P2Y_12_ inhibitor	1,973 (96.1)	1,758 (96.7)	73 (91.3)	74 (91.4)	68 (90.7)	<0.001
Oral anticoagulation	267 (13.0)	238 (13.1)	9 (11.3)	12 (14.8)	8 (10.7)	0.87
ACEI/ARB	1,593 (77.6)	1,430 (78.7)	57 (71.3)	51 (63.0)	55 (73.3)	0.004
Beta-blocker	1,535 (74.7)	1,371 (75.4)	62 (77.5)	52 (64.2)	50 (66.7)	0.047
Statin	1,909 (92.9)	1,702 (93.6)	70 (87.5)	70 (86.4)	67 (89.3)	0.009
Steroid	79 (3.9)	43 (2.4)	1 (1.3)	3 (3.7)	32 (42.7)	<0.001
NSAIDs	49 (2.4)	34 (1.9)	2 (2.5)	0 (0)	13 (17.3)	<0.001

Values are n (%).

ACEI = angiotensin converting enzyme inhibitor; ARB = angiotensin II receptor blocker; NSAID = nonsteroidal anti-inflammatory drug; other abbreviations as in Table 1.

**Table 6:** Clinical Outcomes After Discharge

	All (N = 2,054)	SMuRFs (n = 1,818)	No SMuRFs (n = 80)	Active Cancer (n = 81)	CSIDs (n = 75)	*P* Value
MACE	220 (10.7)	167 (9.2)	9 (11.3)	27 (33.3)	17 (22.7)	<0.001
All-cause death	132 (6.4)	92 (5.1)	6 (7.5)	25 (30.9)	9 (12.0)	<0.001
Myocardial infarction	67 (3.3)	61 (3.4)	1 (1.3)	2 (2.5)	3 (4.0)	0.79
Ischemic stroke	42 (2.0)	31 (1.7)	2 (2.5)	3 (3.7)	6 (8.0)	0.005
Major bleeding events	68 (3.3)	53 (2.9)	3 (3.8)	7 (8.6)	5 (6.7)	0.01

Values are n (%).

Abbreviations as in Tables 1 and 2.

**Central Illustration:**
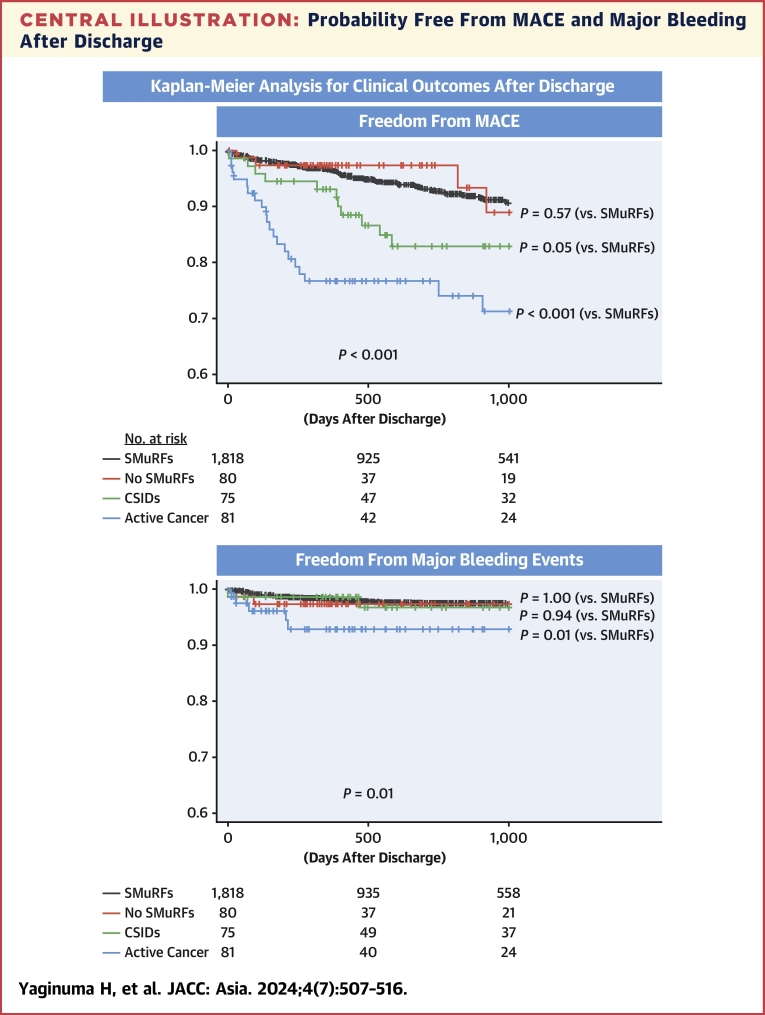
Probability Free From MACE and Major Bleeding After Discharge Kaplan-Meier analysis for MACE and major bleeding events after discharge. Outcomes in the active cancer, CSIDs, and no SMuRFs groups were compared with those in the SMuRFs group. CSID = chronic systemic inflammatory disease; MACE = major adverse cardiovascular event(s); SMuRF = standard modifiable cardiovascular risk factor.

**Table 7:** Cox Proportional Hazards Analysis for MACE After Discharge

	Univariable		Multivariable	
	HR (95% CI)	*P* Value	HR (95% CI)	*P* Value
Age, y	1.03 (1.02-1.05)	<0.001	1.03 (1.02-1.05)	<0.001
Men	1.00 (0.72-1.39)	>0.99	1.18 (0.84-1.65)	0.34
Cardiogenic shock	1.16 (0.77-1.77)	0.48	1.12 (0.73-1.72)	0.59
Drug-eluting stent	0.54 (0.37-0.80)	0.002	0.61 (0.41-0.91)	0.01
Oral anticoagulation	1.19 (0.81-1.74)	0.37	1.09 (0.74-1.60)	0.67
Statin	0.61 (0.42-0.90)	0.01	0.79 (0.53-1.17)	0.24
No SMuRFs group	1.24 (0.63-2.41)	0.54	1.18 (0.60-2.32)	0.63
Active cancer group	3.59 (2.40-5.37)	<0.001	3.04 (2.00-4.62)	<0.001
CSIDs group	1.67 (1.02-2.75)	0.04	1.91 (1.15-3.17)	0.01

Abbreviations as in Tables 1 and 2.

**Table 8:** Cox Proportional Hazards Analysis for Major Bleeding Events After Discharge

	Univariable		Multivariable	
	HR (95% CI)	*P* Value	HR (95% CI)	*P* Value
Age, y	1.03 (1.01-1.06)	0.004	1.03 (1.00-1.05)	0.02
Men	0.50 (0.30-0.82)	0.006	0.57 (0.34-0.96)	0.03
Cardiogenic shock	1.29 (0.62-2.69)	0.50	1.19 (0.56-2.52)	0.66
Drug-eluting stent	0.69 (0.33-1.45)	0.33	0.77 (0.35-1.67)	0.51
Oral anticoagulation	1.52 (0.82-2.84)	0.19	1.43 (0.75-2.71)	0.27
Statin	0.71 (0.34-1.48)	0.36	0.83 (0.39-1.67)	0.51
No SMuRFs group	1.33 (0.42-4.22)	0.63	1.29 (0.40-4.19)	0.67
Active cancer group	3.11 (1.42-6.81)	0.005	2.85 (1.27-6.41)	0.01
CSIDs group	1.48 (0.59-3.70)	0.40	1.57 (0.62-4.00)	0.34

Abbreviations as in Tables 1 and 2.

## Discussion

In the present multicenter registry, >10% of patients with acute MI were grouped as active cancer, CSIDs, and no SMuRFs in total. In-hospital ischemic events occurred more frequently in patients with no SMuRFs than in other groups, whereas a bleeding risk was highest in the active cancer group. After discharge, a long-term MACE risk was higher in the active cancer and CSIDs groups, and the risk of bleeding was highest in the active cancer group. In any scenario, patients with at least one SMuRFs who did not have active cancer and CSIDs had the lowest risk of ischemic and bleeding events during hospitalization and after discharge, suggesting that the 3 patient groups should be taken into account when managing and treating patients with acute MI because of their vulnerability.

### Active cancer, CSIDs, and no SMuRFs in acute MI

A recent large-scale global study confirmed that SMuRFs are major underlying etiologies of the development of atherosclerotic cardiovascular diseases including acute MI, in which an attributable fraction of aggregate SMuRFs for cardiovascular disease, defined as hypertension, diabetes, dyslipidemia, current smoking, and body mass index in the global study, was >50%.^1^ However, a significant proportion of patients with acute MI reportedly have no SMuRFs, ranging from 5% to 25%,^3^^,^^6^ in whom the prognosis after MI was counterintuitively worse than those having SMuRFs.^5^, ^6^, ^7^, ^8^ Among patients with no SMuRFs but developing acute MI, active cancer and CSIDs were potential leading causes of MI,^9^ presumably caused by proinflammatory conditions. Given the poor prognosis of patients with cancer and CSIDs after MI,^10^, ^11^, ^12^, ^13^, ^14^ such patient groups may have different risk profiles from those with “truly” no SMuRFs. In previous studies, the prevalence of active cancer in patients with acute MI was reported to be 2% to 10%.^10^^,^^11^^,^^32^^,^^33^ In addition, CSIDs may account for approximately 5% of patients with acute MI.^12^, ^13^, ^14^ The prevalence of active cancer, CSIDs, and no SMuRFs in the present study were in line with previous literature, indicating that such vulnerable patient populations accounted for >10% in total in acute MI, although data on head-to-head comparisons among the groups are lacking. It may be challenging to biologically compare patients with cancer, CSIDs, and no SMuRFs. Nonetheless, this study addressed the risk profiles of those with “truly” no SMuRFs and compared SMuRFs patients with those with cancer and CSIDs from a clinical perspective.

### Prognostic impact of active cancer, CSIDs, and no SMuRFs

As shown in previous reports, the present study confirmed that patients with active cancer, CSIDs, and no SMuRFs had poor prognosis after acute MI. Importantly, even when excluding patients with cancer and CSIDs, the lack of SMuRFs was associated with worse outcomes, particularly for in-hospital events. Although the underlying mechanisms of increased risk of MACE in patients with acute MI and no SMuRFs remain unclear, potential explanations include the lack of pretreatment and targeted intervention for SMuRFs, and under-reporting of risk factors because of severe clinical presentation in this patient population. Indeed, cardiogenic shock and cardiac arrest were more frequently observed in the no SMuRFs group among the 4 groups. The incidence of in-hospital MACE in patients with no SMuRFs was higher than in other groups, with an approximately 2-fold increased risk of mortality in the no SMuRFs group, probably caused by the severe presentation. Of note, however, the prognosis after discharge in patients with no SMuRFs was similar to that of the SMuRFs group, as was shown in the large-scale SWEDEHEART registry.^5^

Clinical outcomes after acute MI in patients with active cancer were characterized by an increased risk of mortality and major bleeding during hospitalization and after discharge. An administrative database study in the United States, including more than 6.5 million patients with acute MI, showed that the presence of active cancer was associated with an in-hospital major bleeding risk with a similar rate to the present study (ie, 18.4%).^11^ This increased risk of bleeding during hospitalization in cancer patients was confirmed by the multivariable analysis. As shown in our previous study, patients with active cancer also had an increased bleeding risk after discharge^10^ along with high mortality. Even after multivariable adjustment, the presence of active cancer as well as CSIDs, older age, and the use of drug-eluting stents was associated with a higher risk of MACE after discharge, mainly driven by the increase in mortality. We believe physicians should be aware that medications for secondary prevention were less likely to be prescribed in the active cancer group in this study, although it may be challenging to improve outcomes through interventions for cardiovascular systems in this patient population. Because active cancer as well as older age and female gender were indicated as factors associated with bleeding events after discharge, these patient characteristics should be taken into account following acute MI. Additionally, our study results also characterized clinical outcomes of patients with CSIDs after MI. The present study showed that compared with the no SMuRFs and active cancer groups, patients with CSIDs had relatively better in-hospital prognosis. On the other hand, the CSIDs group had high ischemic and bleeding risks after discharge, as secondary to the active cancer group. These findings suggest that patients with CSIDs may require close and long-term follow-up after acute MI, including therapeutic interventions with medications against thrombogenesis and inflammation.^34^ More importantly, in any scenario (during hospitalization or after discharge and in ischemic or bleeding outcomes), patients with at least 1 SMuRFs and no active cancer or CSIDs had the lowest risks of ischemic and bleeding events. We believe that the identification of vulnerable patient groups, including active cancer, CSIDs, and no SMuRFs, may convey better therapeutic strategies and will advance future research in this field of acute MI.

### Study limitations

This was a retrospective, observational study. Thus, our results do not indicate a causal relationship. Given the highly different baseline patient characteristics among the 4 groups (Table 1), pairwise matching analysis was not possible in the present study population. Despite the multicenter setting and large sample size, the number of patients included in the active cancer, CSIDs, and no SMuRFs groups was relatively small. We found a lower-than-expected rate of active cancer and CSIDs among patients having none of SMuRFs (ie, 15.5%). In addition, data on medications on admission and during follow-up were unavailable, and details on the stages and treatment of cancers are missing. Because of the potential that the prevalence of no SMuRFs in acute MI may be lower in East Asia than in Western countries due to ethnicity,^5^, ^6^, ^7^, ^8^ external generalizability of the present study results is unclear, particularly in non-Asian populations.

## Conclusions

Compared with patients who had at least 1 SMuRF, the presence of active cancer and CSIDs and the lack of SMuRFs were characterized by worse ischemic and bleeding outcomes during hospitalization and after discharge in a setting of acute MI, suggesting that the identification and recognition of the 3 patient groups may aid in clinical decision-making after PCI.

Perspectives **COMPETENCY IN MEDICAL KNOWLEDGE:** In patients with acute MI, in-hospital MACE were more frequent in those with no SMuRFs, followed by patients with active cancer and CSIDs. After discharge, MACE and bleeding event rates were higher in patients with cancer and CSIDs compared with those with and without SMuRFs.

**TRANSLATIONAL OUTLOOK:** Given the vulnerability of patients with active cancer, CSIDs, and no SMuRFs, attention should be paid after acute MI when treating. Further clinical investigations and therapeutic approaches are needed in patients with active cancer, CSIDs, and no SMuRFs.

## Funding Support and Author Disclosures

The authors have reported that they have no relationships relevant to the contents of this paper to disclose.
