# Supplementary material for: Impact of Cancer, Inflammation, and No Standard Risk Factors in Patients With Myocardial Infarction
Source: JACC Asia. 2024 May 28;4(7):507–16. doi: 10.1016/j.jacasi.2024.03.008 (PMC11291396; doi:10.1016/j.jacasi.2024.03.008)
Supplement: Supplemental Table S1–S3 [file mmc1.docx]

**Impact of Cancer, Inflammation, and No Standard Risk Factors in Patients With Myocardial Infarction**

**Running title:** Cancer, inflammation, and risk factors in AMI

Hiroaki Yaginuma, MD ^1^; Yuichi Saito, MD ^1^; Hiroki Goto, MD ^1^; Kazunari Asada, MD ^1^; Yuki Shiko, PhD ^2^; Takanori Sato, MD ^1^; Osamu Hashimoto, MD ^3^; Hideki Kitahara, MD ^1^; Yoshio Kobayashi, MD ^1^

**Table S1. Details of cancer**

| Variable | All  (n=2480) |
| --- | --- |
|  |  |
| All cancer | 104 (4.19%) |
| Colon cancer | 18 (0.73%) |
| Blood cancer | 17 (0.69%) |
| Lung cancer | 14 (0.56%) |
| Liver cancer | 14 (0.56%) |
| Prostate cancer | 11 (0.44%) |
| Gastric cancer | 7 (0.28%) |
| Esophageal cancer | 5 (0.20%) |
| Renal cancer | 5 (0.20%) |
| Gynecologic cancer | 4 (0.16%) |
| Pancreatic cancer | 4 (0.16%) |
| Head and neck cancer | 4 (0.16%) |
| Bladder cancer | 3 (0.12%) |
| Bile duct cancer | 2 (0.08%) |
| Duodenal cancer | 1 (0.04%) |
| Gallbladder cancer | 1 (0.04%) |
| Breast cancer | 1 (0.04%) |
| Retroperitoneal tumor | 1 (0.04%) |
| Seminoma | 1 (0.04%) |
| Malignant melanoma | 1 (0.04%) |
| Multiple endocrine neoplasia | 1 (0.04%) |
| Meningioma | 1 (0.04%) |

Some cancers were overlapped with each other.

**Table S2.** **Details of inflammatory disease**

| Variable | All  (n=2480) |
| --- | --- |
|  |  |
| All inflammatory diseases | 94 (3.79%) |
| Rheumatoid arthritis | 39 (1.57%) |
| Systemic lupus erythematosus | 9 (0.36%) |
| Ulcerative colitis | 9 (0.36%) |
| Sjögren's syndrome | 7 (0.28%) |
| Antiphospholipid syndrome | 6 (0.24%) |
| Psoriasis | 5 (0.20%) |
| Systemic sclerosis | 3 (0.12%) |
| Microscopic polyangiitis | 3 (0.12%) |
| Crohn disease | 2 (0.08%) |
| Sarcoidosis | 2 (0.08%) |
| Polymyositis/dermatomyositis | 2 (0.08%) |
| IgG4 related disease | 2 (0.08%) |
| Giant cell arteritis | 1 (0.04%) |
| Granulomatosis with polyangiitis | 1 (0.04%) |
| IgA vasculitis | 1 (0.04%) |
| Primary sclerosing cholangitis | 1 (0.04%) |
| Takayasu arteritis | 1 (0.04%) |
| Autoimmune hepatitis | 1 (0.04%) |
| Cryoglobulinemic vasculitis | 1 (0.04%) |
| Eosinophilic granulomatosis with polyangiitis | 1 (0.04%) |

Rheumatoid arthritis and Sjögren's syndrome, systemic lupus erythematosus and Sjögren's syndrome, and systemic sclerosis and antiphospholipid syndrome were overlapped in one of each case. IgA = immunoglobulin A; IgG = immunoglobulin G.

**Table S3. Fine and Gray regression analysis for major bleeding events after discharge**

| Variable | Multivariable | |
| --- | --- | --- |
|  | HR (95% CI) | p value |
| Age (years) | 1.02 (1.00-1.05) | 0.03 |
| Men | 0.56 (0.34-0.92) | 0.02 |
| Cardiogenic shock | 1.18 (0.54-2.59) | 0.68 |
| Drug-eluting stent | 0.79 (0.36-1.75) | 0.56 |
| Oral anticoagulation | 1.44 (0.78-2.68) | 0.24 |
| Statin | 0.86 (0.38-1.94) | 0.71 |
| No SMuRFs group | 1.24 (0.36-4.21) | 0.73 |
| Active cancer group | 2.37 (1.02-5.48) | 0.04 |
| CSIDs group | 1.54 (0.61-3.88) | 0.36 |

Death was considered as a competing risk. CI = confidence interval; CSIDs = chronic systemic inflammatory diseases; HR = hazard ratio; SMuRFs = standard modifiable cardiovascular risk factors.
